# Identification and validation of *N*-acetylputrescine in combination with non-canonical clinical features as a Parkinson’s disease biomarker panel

**DOI:** 10.1038/s41598-024-60872-3

**Published:** 2024-05-02

**Authors:** Kuan-Wei Peng, Allison Klotz, Arcan Guven, Unnati Kapadnis, Shobha Ravipaty, Vladimir Tolstikov, Vijetha Vemulapalli, Leonardo O. Rodrigues, Hongyan Li, Mark D. Kellogg, Farah Kausar, Linda Rees, Rangaprasad Sarangarajan, Birgitt Schüle, William Langston, Paula Narain, Niven R. Narain, Michael A. Kiebish

**Affiliations:** 1 BPGbio, 500 Old Connecticut Path, Framingham, MA 01701 USA; 2 Department of Pathology, Harvard Medical School, Boston, MA USA; 3 Department of Laboratory Medicine, Boston Children’s Hospital, 300 Longwood Avenue, Boston, MA 02115 USA; 4 Department of Neurology, Weill Institute for Neurosciences, University of California San Francisco, San Francisco, CA 94158 USA; 5 Neurocrine Biosciences, San Diego, CA 92130 USA; 6 Department of Pathology, Stanford School of Medicine, Stanford, CA 94305 USA

**Keywords:** Diagnostic markers, Predictive markers, Neurological disorders, Metabolomics

## Abstract

Parkinson’s disease is a progressive neurodegenerative disorder in which loss of dopaminergic neurons in the substantia nigra results in a clinically heterogeneous group with variable motor and non-motor symptoms with a degree of misdiagnosis. Only 3–25% of sporadic Parkinson’s patients present with genetic abnormalities that could represent a risk factor, thus environmental, metabolic, and other unknown causes contribute to the pathogenesis of Parkinson’s disease, which highlights the critical need for biomarkers. In the present study, we prospectively collected and analyzed plasma samples from 194 Parkinson’s disease patients and 197 age-matched non-diseased controls. *N*-acetyl putrescine (NAP) in combination with sense of smell (B-SIT), depression/anxiety (HADS), and acting out dreams (RBD1Q) clinical measurements demonstrated combined diagnostic utility. NAP was increased by 28% in Parkinsons disease patients and exhibited an AUC of 0.72 as well as an OR of 4.79. The clinical and NAP panel demonstrated an area under the curve, AUC = 0.9 and an OR of 20.4. The assessed diagnostic panel demonstrates combinatorial utility in diagnosing Parkinson’s disease, allowing for an integrated interpretation of disease pathophysiology and highlighting the use of multi-tiered panels in neurological disease diagnosis.

## Introduction

Parkinson’s Disease (PD) is a progressive neurological disorder characterized by motor features including tremors, bradykinesia, muscle rigidity, and postural instability, as well as non-motor features such as loss of sense of smell, REM sleep behavior disorder, and autonomic dysfunctions which can include constipation, urinary problems, changes in heart rate variability, psychiatric disturbances with anxiety, and depression as well as cognitive decline^1^. Pathologically, PD is defined by dopaminergic neuronal loss in the substantia nigra pars compacta (SN), and intracellular inclusions called Lewy bodies (LB) in the neurons of affected brain regions^2,3^. Abnormal handling of misfolded proteins by the ubiquitin–proteasome and the autophagy–lysosomal systems^4,5^, increased oxidative stress, mitochondrial dysfunction, and inflammation, are well-described mechanisms involved in the pathogenesis of PD^5,6^.

The misdiagnosis rate of patients with PD in the clinical setting can be as high as 25–42%^7^ in the early stage of the disease^8–10^. A molecular diagnostic test, which can be used to identify those with early stages of PD is a critical unmet need. Reliable diagnostic biomarkers are essential for identifying populations at risk and those that are pathologically susceptible to disease impetus. This provides an opportunity for an early and accurate diagnosis to predict the disease occurrence and progression. The diagnostic biomarker can be used as an objective tool to characterize evaluation indicators stratifying normal and pathogenic biological processes^11^.

Significant progress has been made in uncovering the complex molecular mechanisms exploited in the pathogenesis of PD. The emergence of several omics techniques, such as transcriptomics, proteomics, and metabolomics, have played a key role in identifying novel pathways associated with dopaminergic neurodegeneration, global system physiological changes, and subsequently PD, which include mitochondrial and proteasomal function as well as synaptic neurotransmission^10^. Additionally, these unbiased techniques, particularly in the brain regions that are uniquely associated with the disease, have greatly enhanced our ability to identify novel pathways, such as axon-guidance, potentially involved in PD pathogenesis^12,13^. To date, there has been extensive focus on the genetic etiology of disease^3,14^. In contrast, multi-omic analysis provides broader connectivity to adaptive and environmental sequalae that drive disease phenotypic effectors. Metabolomics assessment, which comprises the broadest capture of integrated biochemical assessment using mass spectrometry or NMR technology, provides a comprehensive view of metabolites tied to the biological phenotype.

Interestingly in PD patients, putrescine levels are increased in cerebrospinal fluid (CSF), whereas the concentration of spermidine is reduced compared to controls^15^. Putrescine is a polyamine that belongs to the category of ubiquitous small polycations that ionically bind to various negatively charged molecules and have many functions, mostly linked to cell growth, survival, and proliferation^15^. Examples of polyamines are putrescine, spermidine and spermine, whose levels are stringently regulated in the human body. Based on partial polyamine data previously reported^16^ and our metabolomics platform outcome, we investigated a prospective PD cohort clinically and evaluated polyamine metabolic changes analytically. Further, we integrated clinical features identified by our Bayesian analysis to be causally associated with clinical outcomes. The use of these clinical features as a phenotypic readout complimented the molecular biomarker analysis. The combination of the CLIA validated assay of *N*-acetylputrescine (NAP) and non-canonical clinical features of Hospital Anxiety and Depression Scale (HADS), smell (BsitTotal), and REM Sleep Behavior Disorder Single-Question Screen score (RBD1Q) demonstrated diagnostic utility^17–19^. This panel might provide broad utility for PD diagnosis and represents the integration of both clinical and molecular presentation of the disease.

## Materials and methods

### Materials

*N*-acetylputrescine reference standard, Bovine Serum Albumin (BSA), Trichloroacetic acid (TCA), Formic Acid (FA) and Isobutyl Chloroformate (IBCF) were obtained from Sigma (St. Louis, MO, USA). Optima LC/MS Grade of Acetonitrile, Water, and Sodium carbonate were purchased from Fisher Scientific (Pittsburgh, PA USA). Human K_2_-EDTA Plasma was supplied by BIOIVT (Westbury, NY, USA).

### Plasma sample collection

K_2_-EDTA plasma samples and clinical data from PD patients and controls were obtained from Parkinson’s Institute and Clinical Center (Sunnyvale, CA., USA). All study subject information shall remain de-identified. Samples were collected within the following range of self-reported fasting time: not less than 4 h, not more than 8 h. Non-diseased controls (n = 199) and Parkinson’s Disease patients (n = 201) were analyzed, with Hoehn and Yahr (H&Y) average scale of 2.1 and Unified Parkinson’s Disease rating scale (UPDRS) score from 10 to 115 (Table 1). The non-diseased control group included 125 males and 72 females. Non-diseased controls were characterized and exhibited no PD symptoms to allow for age matched controls. The PD patient cohort included 103 males and 91 females. All volunteers participating in this study gave their informed consent for inclusion before they participated in the study. Research use of the samples was conducted by the terms outlined within the informed consent form and the terms set forth therein, along with the tenets of the Declaration of Helsinki and its later amendments or comparable ethical standards. Informed consent was obtained from all the participants in the study NCT02016095 and all procedures and protocols were approved by the El Camino Hospital IRB, Mountain View, protocol ECH-10-17 and the Parkinsons Disease Institute according to guidelines. The diagnosis for PD was determined by a movement disorder specialist using Hoehn and Yahr diagnostic criteria, UK brainbank criteria, and Unified Parkinson's Disease Rating Scale (UPDRS). Additional scales for determining supporting clinical symptoms included sense of smell (Brief smell identification test), hospital anxiety and depression scale (HADS), Montreal cognitive assessment (MoCA), and REM sleep behavior disorder (RBD) question.

**Table 1:** Patient Demographics.

	Non-disease	Parkinson’s disease
Total patients	197	194
Male	125	103
Female	72	91
Age, mean years (SD)	65.4 (9.6)	64.7 (9.1)
H&Y stage		
Mean ± SD	N/A	2.1 ± 0.7
Case (n)	N/A	I(43), II(127),III (22), IV (7), V(1)
UPDRS mean, (SD), (range)	N/A	40.4, (16.9), (10–115)
UPDRS-III mean, (SD), (range)	N/A	25.6, (11.0), (1–69)

### Sample preparation of NAP in plasma extraction and derivatization

Solutions and reagents were brought to room temperature (RT) before initiating the extraction process. Standards (STD), sub-stocks, quality controls (QCs), surrogate matrix, and unknown human plasma samples were thawed at RT. Two double blanks were included in each batch by transferring 50 µl of 2.5% BSA into clean 2 ml microcentrifuge tubes. STD, QCs, and unknowns (50 µl) were added to separate microcentrifuge tubes. Liquid chromatography mass spectrometry grade water (100 µl) and 4% TCA (100 µl) were added to these tubes and vortexed for 1 min on a multi-tube vortex mixer (VWR International LLC, Radnor, PA).

Samples were centrifuged (Eppendorf 5415D) for 15 min at 10,000×*g* a 5 °C. To a clean microcentrifuge tube, 200 µl of LCMS grade water and 125 µl 1 M Sodium carbonate buffer were added. Each sample (125 µl) was transferred to a microcentrifuge tube containing LCMS grade water and 1 M sodium carbonate buffer. To these tubes, 25 µl isobutyl chloroformate was added. All samples were vortexed for approximately 1 min in a multi-tube vortex. The samples were then incubated for 15 min at 35 °C and shaken at 150 rpm. Next, the samples were placed in the centrifuge for 10 min at 10,000×*g* at 5 °C. After removing from the centrifuge, the samples were extracted using SPE cartridges (Waters Oasis HLB 10 mg 1CC Cartridge) in conjunction with the UTC Positive Pressure manifold. Using low positive pressure, the columns were conditioned in the following sequence: Add 1 ml of Methanol and wait until liquid flowed through, next add 1 mL of LCMS grade water and wait until the liquid flowed through. Finally, samples (275 µl) were loaded onto the cartridge. Samples sit for approximately 1 min or until the sample flowed through entirely. Low positive pressure was applied to remove any residual liquid. Samples were eluted by adding 250 µl 80:20:0.1 ACN:H2O:FA to each cartridge. Samples flow through with gravity, approximately 2 min, before using low positive pressure to elute the remaining liquid into clean microcentrifuge tubes. The eluant was dried under a gentle stream of N_2_ using a Turbovap at 37 °C. All samples were reconstituted by adding 200 µl of Reconstitution Solution (10:90:0.1, ACN:H2O:FA) and vortexed for 1 min. Samples were then transferred to amber glass HPLC vials with 0.3 ml inserts. Samples were loaded directly for injection onto the LC–MS/MS, or the extracts were stored at 4 °C until injection.

### LC–MS/MS (MRM) analysis for NAP

The multiple reaction monitoring (MRM) analyses was performed on an AB SCIEX QTRAP^®^ 5500 mass spectrometer (SCIEX) equipped with an electrospray source, Shimadzu (Kyoto, Japan) Ultra-Fast Liquid Chromatograph (UFLC) (LC-20AD XR pumps and SIL-20AC XR autosampler), and a Poroshell 120, EL-C18, (2.1 × 50 mm, 2.7 µ) column (Agilent, Santa Clara, CA). The MRM of derivatized *N*-Acetyl putrescine precursor and transition were m/z 231.00 and m/z 115.00, respectively, used as quantifier (Suppl. Table 1). Liquid chromatography was carried out at a flow rate of 0.350 mL/min, and the sample injection volume was 5 µL. The column was maintained at a temperature of 40 °C. Mobile phase A consisted of 0.1% Formic Acid (Sigma Aldrich) in water (Fisher Scientific), and mobile phase B consisted of 0.1% formic acid in acetonitrile (Fisher Scientific). The gradient with respect to %B was as follows: 0–4 min, 5%; 4–5.5 min, 40%; 5.5–6.1 min, 95%; 6.1–10.0 min 5%. The instrument parameters for the 5500 QTRAP mass spectrometer were as follows: ion spray voltage of 5500 V, curtain gas of 20 psi, collision gas set to “medium”, interface heater temperature of 500 °C, nebulizer gas (GS1) of 40 psi, and ion source gas (GS2) of 40 psi and unit resolution for both Q1 and Q3 quadrupoles. Data analysis was performed using the AB SCIEX Analyst® software (version 1.5.1 or 1.6.2, Sciex, Framingham, MA), and peak integrations were reviewed manually.

### Identification of NAP and clinical features as a potential PD biomarker panel

A multi-omic assessment of plasma was performed as previously reported^20^. The statistical analysis was conducted using R-Studio (2020, Version 3.6.2). Logistic regression was used to build all the models in ROC analysis. The selection of clinical variables was based on causal graphs (networks) generated by bAIcis^®^, which relies on Bayesian network methods to learn from directed acyclic graphs^21^. To identify potential causal drivers of the PD status, an ensemble model from all variables was generated using bAIcis^®^, and the clinical variables directly connected to the outcome of interest were selected for further exploration. Variables of the best combination of multivariate models were chosen based on balancing the AUC and the complexity of the model. The 95% confidence interval was computed with 2000 stratified bootstrap replicates. Differences in means between PD and non-PD controls were assessed using t-test. Statistical significance for all analyses was determined at *p* < 0.05.

## Results

### General demographics and discovery assessment

**Figure 1:**
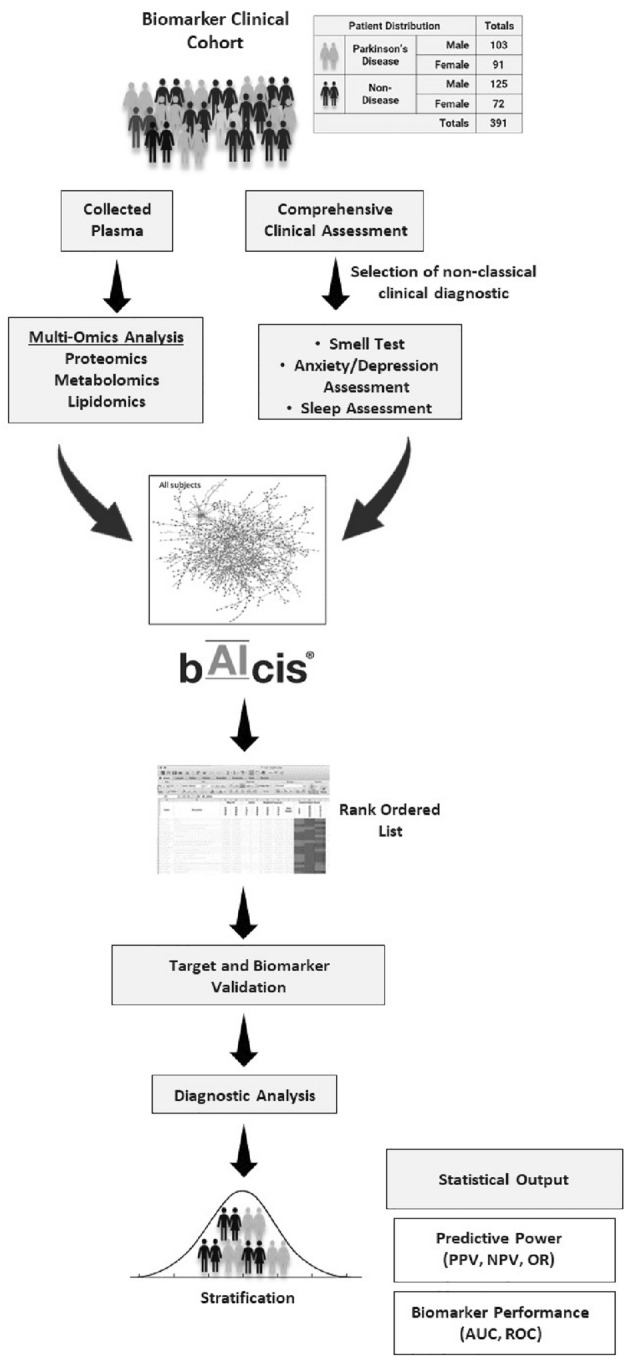
Study design. Biomarker Discovery Pipeline and Study Design. Single center observational study to assess markers in Parkinson’s patients and non-disease controls. Multi-omics analysis was performed and CLIA validated procedures were subsequently employed for quantitative biomarker assessment.

A total of 194 PD patients and 197 non-disease controls participated in this study, with an average age of 65.4 and 64.7 years old, respectively (Table 1). Of the 194 PD patients, 139 PD patients had medical information to Levodopa/Carbidopa administration including 90 PD patients (dosage range: 50–2000 mg, mean: 338 mg). The average score of Hoehn and Yahr (H&Y) scale was 2.1, including the 43 PD patients for stage I, 127 for stage II, 22 for stage III, 7 for stage IV, and 1 for stage V. The Unified Parkinson Disease rating scale (UPDRS) score for these PD patients ranged from 10 to 115. Metabolomic, lipidomic, and proteomic assessment of plasma samples were performed and integrated with statistical, Bayesian analysis and regression assessment of clinical and molecular features, which identified a single metabolite and was further developed in a CLIA lab for regulated bioanalysis (Fig. 1).

### Assay development and validation

To quantify NAP in human K_2_-EDTA plasma, the quantification method using LC–MS/MS was developed. Due to low circulating levels of NAP, the sensitivity of quantification was improved using derivatization of isobutyl chloroformate in the sample preparation^22,23^. The validation performance of the NAP assay is summarized in Suppl. Tables 1–8, including linearity, precision, matrix effect, system suitability, short-term stability, long-term stability, and reproducibility in the autosampler. Fit-for-purpose method validation results demonstrated quantitative ranges for NAP from 1 to 85 ng/mL in plasma analysis (Suppl. Table 3). The results of validation assessed by QCs met acceptance criteria (Suppl. Tables 2–8).

### Human plasma sample analysis of NAP

**Figure 2:**
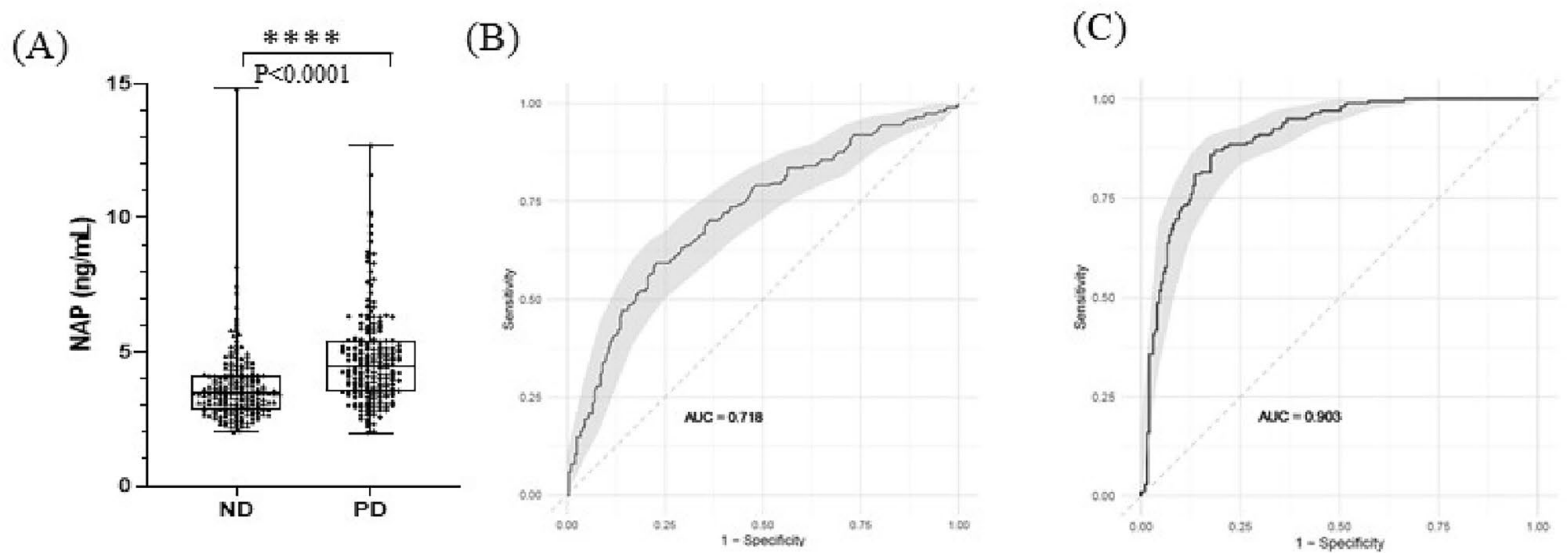
Plasma Sample Analysis for NAP and Receiver Operation Characteristic (ROC) Curve Analysis (**A**) The plasma levels of NAP between non-disease and PD cohort.; ROC curve analysis for NAP alone (**B**) ROC curve analysis for NAP alone and (C) ROC curve analysis for NAP plus three clinical variables (C95% confident interval (CI) using Bootstrapping approach in ROC curve. Statistics was calculated by t-test, statistically significant: ****p < 0.0001.

Utilizing our CLIA validated quantification method, the K_2_-EDTA plasma samples from a total of 391 participants, including 197 non-disease (ND) and 194 PD cohort, were analyzed for NAP quantitation. When comparing PD in male and female patients, there were no statistical differences of plasma levels of NAP in gender that was observed (one-way ANOVA, *p* = 0.6492) (Data not shown). A significant difference in plasma NAP between ND and PD was detected (t-test, *p* < 0.0001). The mean concentrations of NAP in the plasma for ND and in the PD cohorts were 3.70 ng/ml and 4.74 ng/ml, respectively (Fig. 2).

### Receiver operating characteristic curve (ROC) analysis of NAP and three clinical features for PD diagnosis

To examine the clinical performance, a receiver operating characteristic curve (ROC) analysis was applied for PD diagnosis in this project. In results of ROC analysis using the trapezoidal rule, the area under the curve (AUC) for NAP alone was 0.72, suggesting that plasma NAP levels demonstrate utility for PD diagnosis. The PD diagnosis of NAP alone showed the specificity as 90% and sensitivity as 35% with a cutoff value of 6.91 ng/ml, respectively. In multivariate logistic regression models, the AUC values using the demographic factors of age, gender, or a combination with NAP were not improved in the separation of ND and PD cohorts (data not shown). Analysis of 121 clinical variables using the bAIcis platform identified smell test (B-SIT), depression and anxiety assessment test (HADS), and acting out dreams test (RBD1Q) as three potential clinical features for diagnosis of PD^17–19^. B-sit assessment demonstrated an average measurement of 10.2 in non-disease participants compared to 7.1 in PD participants. HADS DTOTAL assessment demonstrated mean values of 1.8 in non-disease participants compared to 3.8 in PD participants. Additionally, RBDNO scores were 91% yes in non-disease participants and 56.7% in PD participants. The multivariate model integrated with NAP and three clinical features revealed an optimal AUC of 0.90 to distinguish the PD from the non-disease cohort with a specificity of 95% and sensitivity of 52%. In predictive proficiency of PD diagnosis, the positive predictive value (PPV) and negative predictive value (NPV) for NAP alone were 0.78 and 0.58, and for the multivariate model, they were 0.91 and 0.66, respectively (Table 2). Additionally, NAP was not associated with increasing Hohn and Yahr scoring or UPDRS scores. Further, NAP did not correlate with current PD medications and patients that had not received any disease modifying medications maintained a statistical increase in NAP, especially with comparison with MAO-B (Supplemental Figs. 1 and 2).

**Table 2:** Summary table for clinical performance of marker panel alone and combination.

Variates	AUC	Specificity	Sensitivity	PPV	NPV	OR
NAP	0.72	0.90	0.35	0.78	0.58	4.79
Smell (BsitTotal)	0.85	0.90	0.66	0.87	0.72	17.4
Depression (HADsDTotal)	0.74	0.95	0.19	0.8	0.54	4.58
Nightmare (RBDNO)	0.67	0.95	0.24	0.83	0.55	5.98
NAP + smell + depression + nightmare	0.90	0.95	0.52	0.91	0.66	20.4

*AUC* area under curve, *PPV* sensitivity, specificity, positive predictive value, *NPV* negative predictive valve, *OR* odds ratio.

## Discussion

Our study is the largest metabolomic biomarker study that identified a combination of clinical non-motor measures (smell, anxiety/depression, and RBD) together with a plasma metabolite NAP with a specificity of 95% and sensitivity of 52%. It is also the first study that includes sufficient data for the calculation of diagnostic performance. PD is the second most common neurodegenerative disease affecting millions of people in the USA and many more worldwide. PD is estimated to occur in about 1% of the population over 60 and 4% of the individuals over 80 years old^24,25^. It is difficult to accurately determine the precise prevalence of PD since the numbers do not include the majority of undiagnosed or misdiagnosed cases. The annual costs incurred for PD in the United States have been estimated to be nearly $11 billion, including $6.2 billion in direct costs^26^. The most significant proportion of cost for PD treatment occurs in the later stages of the disease when symptoms are most severe^27^. Any diagnostic tool that could help identifying patients potentially earlier for this disease along with an effective therapeutic strategy that could halt PD symptoms in the prodromal disease stages with no further progression would greatly reduce disease burden for patients and families. In parallel, there is a critical need to develop diagnostic biomarkers for early disease detection using combinatorial PD biomarkers of clinical signs and blood metabolites.

Comprehensive understanding of human health and disease requires interpretation of complex biological processes at multiple levels such as genome, epigenome, transcriptome, proteome, and metabolome. These together can be classified as “multi-omics” data. The availability of multi-omics data has advanced the field of medicine and biology^28^. In this study, we have taken an integrative approach that has combined multi-omics data in order to highlight the interrelationships of the involved biomolecules and their clinical phenotype in disease^29^.

In three recent PD biomarker metabolomic analyses, several altered metabolites were identified, including amino acids, acylcarnitines, and polyamines in PD, however, these studies did not utilize CLIA-validated assays and were underpowered (Table 3). This first study used urinary metabolomic profiling of 18 metabolites, most of these branched chain, tryptophan, and phenylalanine amino acids, demonstrated discrimination capability in early, mid, and advanced stage PD patients (ND = 65; PD = 92)^30^. A major pitfall of the use of amino acids as biomarkers is that they could be overrepresented due to unbalanced demographics within the cohort or influence from concomitant medications. The second study assessed plasma metabolomic profiling and found acylcarnitines related to mitochondrial beta-oxidation as potential early diagnostic PD biomarkers. A group of long-chain acylcarnitines (AC12-14) (ND = 32,45; PD = 109,145) and nine fatty acid (8–18) metabolites (ND = 40; PD = 41) were found to be present in early stages of PD diagnosis^31,32^. Due to the possible influence of other comorbidities within these groups, the use of acyl carnitines became a challenge for diagnosis. Moreover, increasing evidence suggests the possible role of polyamines in PD underlined several potential PD biomarkers in the mechanism of polyamine synthesis and metabolism^16,33^. In a small scale clinical study (n = 33), putrescine and N1-Acetylspermidine in cerebrospinal fluid (CSF) were significantly higher in PD patients compared to the control group^16^. Furthermore, the plasma level of N8-acetylspermidine and N1, N8-diacetylspermidine demonstrated significant accuracy in ROC curve analysis and positively correlated with H&Y stages^33^. Although these reports mentioned elevated NAP levels of plasma in PD patients, no further precise details of NAP clinical performance were elucidated. However, these measurements were not confirmed on robust bioanalytical platforms^33,34^. Additional studies have also evaluated broad metabolomic as well as targeted polyamine analysis in CSF as well as red blood cells, demonstrating metabolic alterations and integrated changes in polyamine metabolism in PD as well as other neurological disorders^33,35,36^. This consistently highlights the systemic alterations in key metabolomic pathways connected with the potential pathogenesis of PD patients in both treated and untreated cohorts. Although we did not see broad polyamine alterations in our study, there are definitely components of this pathway that have consistently been found to be altered.

**Table 3:** Summary of PD Biomarker Discovery from Published Literatures.

Metabolite biomarkers					
References	Man-Jeong Paik et. al (2010)	Hemi Luan et. al (2015)	Florence Burte et. al (2017)	Shinji Saiki et al. (2019)	BPGbio (2023)
Markers	N1-acetylspermidine, and putrescine spermidine	Acetylphenylalanine and other 45 metabolites	Serum Oxalate and other 19 metabolites	N1,N8 diacetylspermidine and spermine/spermidine ratio	NAP and 3 clinical covariates
Assay Method	GC–MS	(LC–MS; GC–MS)	(LC–MS; GC–MS; BDNF ELISA)	(LC–MS/MS)	(LC–MS/MS)
Invasiveness	Moderately invasive (CSF)	Low (Urine)	Minimally invasive (serum)	Minimally invasive (plasma & serum)	Minimally invasive (plasma)
Population Size	ND:24; PD:9	ND:65; PD:92	ND:40; PD:41	(ND:45; PD:145); (ND 49; PD186)	ND:197; PD:194
ROC Analysis	Insufficient data	AUC 0.65–0.97	AUC 0.72–0.85	AUC:0.95	AUC:0.90
Diagnostic Performance	Insufficient data for SE, SP, PPV, NPV	Insufficient data for SE, SP, PPV, NPV	Insufficient data for SE, SP, PPV, NPV	Insufficient data for SE, SP, PPV, NPV	SE: 52%; SP: 950%; PPV 91%; NPV 66%,
CLIA/GLP Validation	NO	NO	NO	NO	YES

*AUC* area under curve in ROC analysis, *SP* sensitivity, *SE* specificity, *PPV* positive predictive value, *NPV* negative predictive valve.

Some limitations in our study are listed as follows. While PD patients enrolled were not assessed by PET or SPECT imaging for the dopamine transporter (DAT) they were clinically diagnosed by a movement disorder specialist. Other illnesses and medication usage by patients may affect the status of their metabolism. Further validation of the proposed biomarker panel in more extensive, well-defined patient cohorts could be the next step to validate the finding of this study.

Lacking standardized criteria supporting PD diagnosis at the prodromal stage, current diagnosis of PD relies primarily on clinical history and neurological assessment by a movement disorder specialist and exclusion of other neurodegenerative diseases. The non-motor clinical signs and symptoms identified from our bAIcis^®^ platform may appear in prodromal PD stage, including olfactory dysfunction, rapid eye movement sleep behavior disorder (RBD), and depression and anxiety^37,38^. The multivariate logistic regression model with NAP and three clinical features demonstrated promising diagnostic performance. This study presents a potential avenue for clinical diagnostics while also underlining a possible role in polyamine metabolism for deciphering elusive etiology of PD.

In this study, we have completed the largest metabolite investigated biomarker study that was assessed using a CLIA validated assay and to identify complementary clinical features providing an AUC = 0.9 and a PPV of 0.91. The multivariate marker model, consisting of three clinical features integrated with NAP, significantly improved the diagnostic accuracy for PD, demonstrating a higher positive predictive value than unintegrated individual variables. Results from this study demonstrated the power of a combined approach to PD biomarker discovery along with the possibility of implementing NAP into clinical biomarker tests.

### Supplementary Information

Supplementary Tables.

Supplementary Figures.

## Data Availability

The datasets generated during and/or analyzed during the current study are not publicly available but are available from the corresponding author on reasonable request.
