# Supplementary material for: Identification and validation of N-acetylputrescine in combination with non-canonical clinical features as a Parkinson’s disease biomarker panel
Source: Sci Rep. 2024 May 2;14:10036. doi: 10.1038/s41598-024-60872-3 (PMC11063140; doi:10.1038/s41598-024-60872-3)
Supplement: Supplementary file 2 — Supplementary Figures. [file 41598_2024_60872_MOESM2_ESM.pptx]

## Slide 1
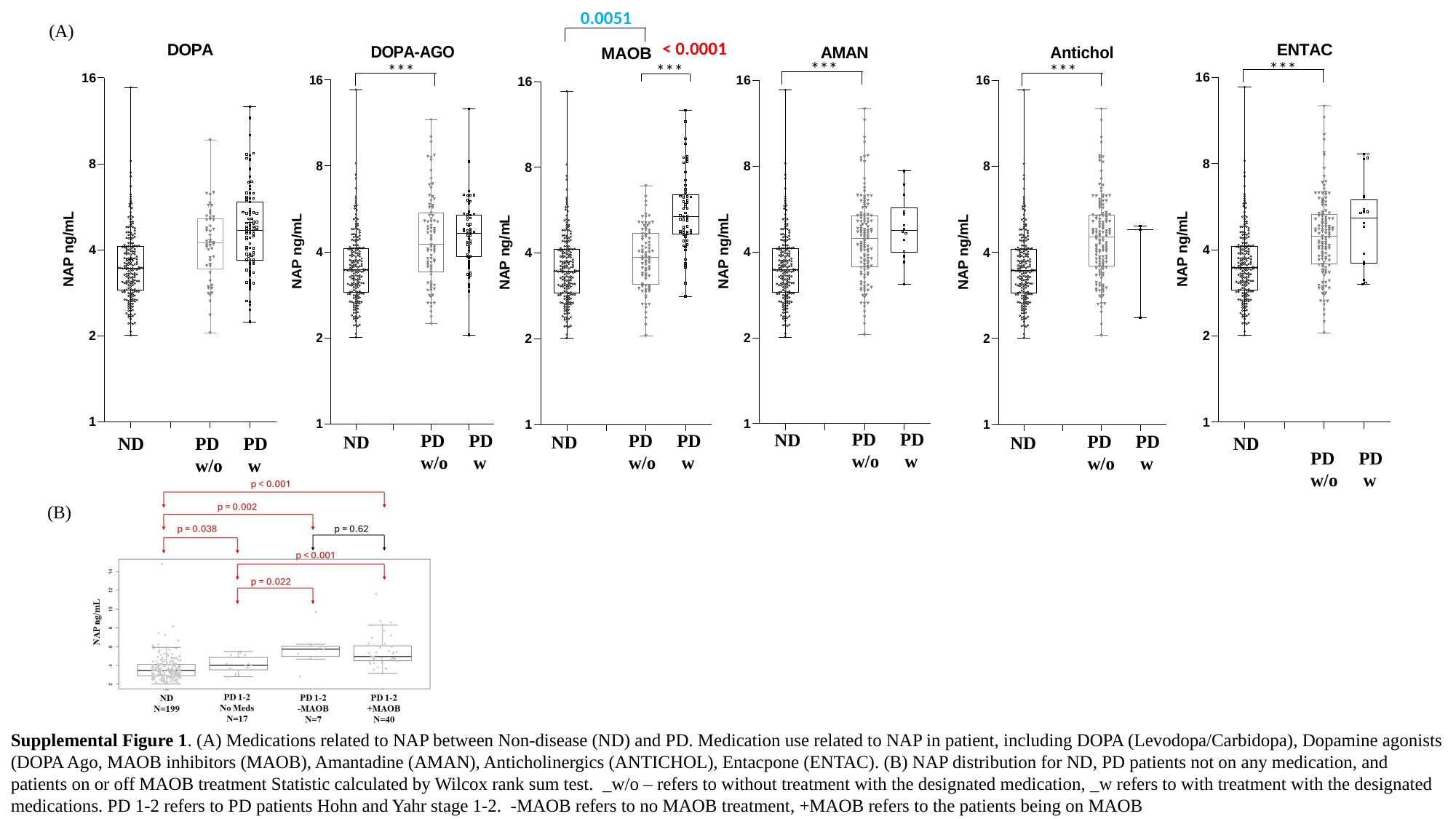

0.0051
(A)
< 0.0001
***
***
***
***
***
PD
w/o
PD
 w
ND
PD
w/o
PD
 w
PD
w/o
PD
 w
PD
w/o
PD
 w
ND
ND
ND
ND
PD
w/o
PD
 w
ND
PD
w/o
PD
 w
(B)
Supplemental Figure 1. (A) Medications related to NAP between Non-disease (ND) and PD. Medication use related to NAP in patient, including DOPA (Levodopa/Carbidopa), Dopamine agonists (DOPA Ago, MAOB inhibitors (MAOB), Amantadine (AMAN), Anticholinergics (ANTICHOL), Entacpone (ENTAC). (B) NAP distribution for ND, PD patients not on any medication, and patients on or off MAOB treatment Statistic calculated by Wilcox rank sum test. _w/o – refers to without treatment with the designated medication, _w refers to with treatment with the designated medications. PD 1-2 refers to PD patients Hohn and Yahr stage 1-2. -MAOB refers to no MAOB treatment, +MAOB refers to the patients being on MAOB

## Slide 2
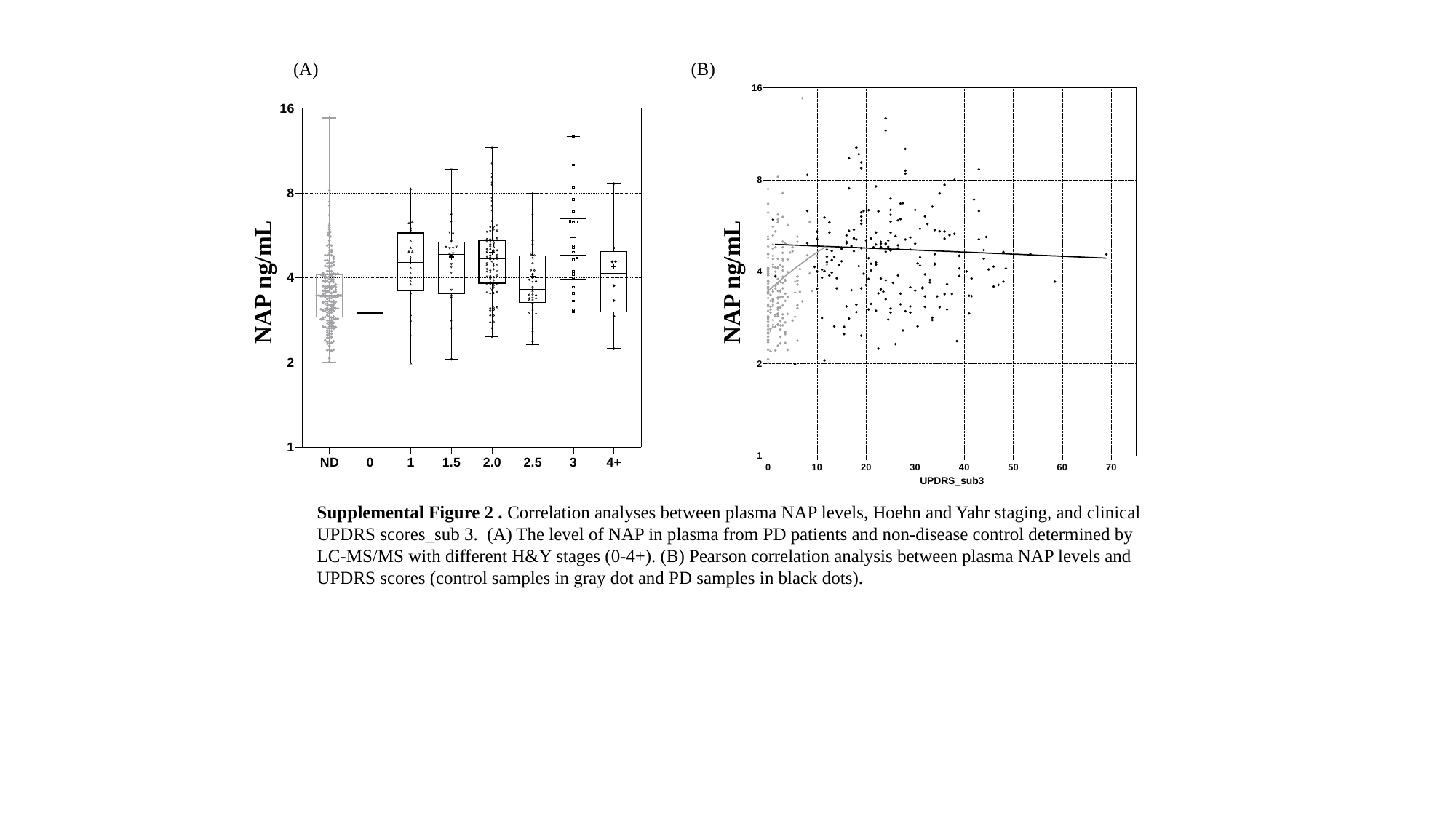

(A)
(B)
NAP ng/mL
NAP ng/mL
Supplemental Figure 2 . Correlation analyses between plasma NAP levels, Hoehn and Yahr staging, and clinical UPDRS scores_sub 3. (A) The level of NAP in plasma from PD patients and non-disease control determined by LC-MS/MS with different H&Y stages (0-4+). (B) Pearson correlation analysis between plasma NAP levels and UPDRS scores (control samples in gray dot and PD samples in black dots).
